# Potential Cost-Effectiveness of Maternal Influenza Immunisation in Low-Income Countries: An Explorative Modelling Study and Value of Information Analysis to Guide Future Clinical Research

**DOI:** 10.3390/vaccines12030232

**Published:** 2024-02-23

**Authors:** Yingying Wang, Michelle L. Giles, Natalie Carvalho

**Affiliations:** 1 Melbourne School of Population and Global Health, University of Melbourne, Parkville 3010, Australia; natalie.carvalho@unimelb.edu.au; 2 Department of Infectious Diseases, University of Melbourne, Parkville 3010, Australia; michelle.giles@unimelb.edu.au; 3 Department of Obstetrics and Gynaecology, Monash University, Clayton 3168, Australia

**Keywords:** maternal immunisation, influenza, cost-effectiveness analysis, value of information analysis, low-income countries, preterm birth, non-specific effects

## Abstract

Maternal influenza immunisation (MII) is recommended for protecting pregnant women and infants under six months of age from severe disease related to influenza. However, few low-income countries have introduced this vaccine. Existing cost-effectiveness studies do not consider potential vaccine non-specific effects (NSE) observed in some settings, such as reductions in preterm birth. A decision tree model was built to examine the potential cost-effectiveness of MII in a hypothetical low-income country compared to no vaccination, considering possible values for NSE on preterm birth in addition to vaccine-specific effects on influenza. We synthesized epidemiological and cost data from low-income countries. All costs were adjusted to 2021 United States dollars (USD). We considered cost-effectiveness thresholds that reflect opportunity costs (USD 188 per disability-adjusted life year averted; range: USD 28–538). Results suggest that even a small (5%) NSE on preterm birth may make MII a cost-effective strategy in these settings. A value of information analysis indicated that acquiring more information on the presence and possible size of NSE of MII could greatly reduce the uncertainty in decision-making on MII. Further clinical research investigating NSE in low-income countries may be of high value to optimise immunisation policy.

## 1. Introduction

### 1.1. Maternal Influenza Immunisation

Pregnant women and infants under six months of age are at an increased risk of severe complications from influenza infection, such as influenza-attributable hospitalisation and death [1,2,3,4]. Global estimates from a 2018 report found approximately 110 million influenza episodes among children aged under five years, with infants under six months constituting about 23% of these cases [5]. While infants under six months are at a higher risk for severe outcomes from influenza compared to older children, they are unable to be vaccinated because no vaccines are currently licensed for this age group [6,7,8]. Maternal influenza immunisation (MII) is an effective and safe strategy to reduce the risk of seasonal influenza and maternal complications from infection, and it is the only way to confer protection to the newborn against seasonal influenza infection through transplacental antibodies [1,2,3,9]. The World Health Organization (WHO) has identified pregnant women as one of the highest priority groups for seasonal influenza vaccination [7,9], and recommends that pregnant women should receive the influenza vaccination at any time during pregnancy [3,10]. Evidence from both high-income countries (HICs) and low- and middle-income countries (LMICs) have demonstrated the efficacy of MII in terms of preventing seasonal influenza, with vaccine efficacy of 35–70% for pregnant women and 28–61% for infants under 6 months of age [3,10,11].

### 1.2. Non-Specific Effects of Vaccines

Vaccines can have both specific and non-specific effects (NSE). Vaccine-specific effects refer to the direct protection conferred by a vaccine against the specific pathogen it was designed to prevent. NSE refers to the additional effect of vaccines on unrelated diseases or infections beyond their specific pathogen design, with an accumulated literature suggesting significant implications for paediatric health, particularly in low-income countries (LICs) [12,13,14]. Recent evidence from randomized controlled trials (RCTs) and observational studies in LICs have demonstrated that some live vaccines, such as measles and Bacile Calmette-Guerin (BCG), not only reduce all-cause childhood mortality but also offer protection against conditions like sepsis [12,13,14].

There is mixed evidence around whether MII offers NSE. Several observational studies from HICs and middle-income countries (MICs) have reported an association between MII and a reduction in preterm birth (PTB) [15,16,17,18,19,20,21,22]. Two large population-based cohort studies from Australia and Canada found a significantly lower risk of PTB in the influenza-vaccinated group compared with the unvaccinated group (adjusted risk ratios of 0.69 and 0.75, respectively) [15,17]. Three other cohort studies in South Africa, Lao PDR and Nicaragua also reported that vaccinated pregnant women were 34% to 44% less likely to develop PTB than unvaccinated pregnant women [16,19,20]. Although a cautious interpretation of findings is required due to potential bias in these studies, it is worth highlighting that in some large cohort studies the NSEs on PTB were found both inside and outside the seasonal influenza season, and the protection was still observed after adjusting for the immortal time bias that can lead to an overestimation of the vaccine effect [17,23]. The available evidence, however, remains mixed. A pooled study of three RCTs conducted in LMICs (Mali, Nepal, South Africa) did not observe any association between MII and PTB [24]. These studies were not designed to evaluate NSEs as the primary health outcomes. This differs from the earlier mentioned observational studies, which were explicitly designed to explore NSEs. This leads to considerable challenges in interpreting discrepancies across existing evidence, as various factors like methodology, sample size, and residual confounders may affect our understanding of MII’s influence on birth outcomes. The mechanisms underlying these NSEs are not yet fully understood [25].

### 1.3. MII Introduction in LICs

In LMICs, vaccination policy and investment decisions are usually guided by a preference for vaccines establishing mortality benefits. However, the hesitancy of policymakers in these regions is often fuelled by uncertainty surrounding the burden of disease, a situation compounded by inadequate surveillance systems [26,27]. Gavi, the Vaccine Alliance, is an international organization that provides funding for vaccine programs in eligible LMICs to improve the equitable and sustainable use of vaccines. According to the WHO vaccine procurement database in 2022, nearly 99% of vaccines in LICs were funded by Gavi. Nevertheless, there was no procurement record of seasonal influenza vaccine from LICs, regardless of sponsorship [28]. Currently, few LICs are listed within the WHO immunization data portal as having implemented MII [29]. In a 2018 Gavi vaccine investment ranking, MII was not shortlisted, receiving lower points in the domains of health and economic impact as compared to the shortlisted candidates (e.g., maternal respiratory syncytial virus vaccine) [30]. This ranking considered the direct effects of MII on influenza-related outcomes only, which may underestimate the potential health and economic benefits of MII [30] and contrasts with WHO recommendations that immunisation frameworks should consider indirect vaccine effects [10,30].

### 1.4. Current Health Economic Evidence from LMICs

Health, economic and financial impacts are key considerations for the implementation of MII and are used by external donors to consider eligibility when developing a new vaccine investment strategy [27,31]. However, current evidence on the health economic evidence of MII is limited in LMICs, particularly LICs [26,32]. According to a systematic review, there has only been one relevant study from LIC (Mali) and it showed that MII in Mali was unlikely to be cost-effective from a societal perspective, with a base incremental cost-effectiveness ratio (ICER) of USD 857 [33,34]. Compared with LICs, MII introduction is more likely to be cost-effective in MICs due to relatively greater healthcare resources and better infrastructure. Moreover, these countries have much higher cost-effectiveness thresholds (CETs), further enhancing cost-effectiveness [34,35,36,37]. A study from South Africa found that the MII introduction for approximately 300,000 pregnant women was cost-effective with an ICER of USD 2010 from a healthcare system perspective and was dominating from a societal perspective [36]. Another cost-effectiveness study on the national influenza immunisation program in Lao PDR also concluded that the MII strategy, which covered 160,000 women per year, was cost-effective with an ICER of USD 5295 [37]. The sensitivity analyses in these three studies all revealed that vaccine-specific effect, disease burden of influenza and vaccine-related costs were the most sensitive inputs [33,36,37]. However, these studies did not consider potential NSEs on adverse pregnancy outcomes.

### 1.5. The Aim of the Study

Adverse pregnancy outcomes disproportionately affect LMICs, with over two thirds of PTB occurring in sub-Saharan Africa and South Asia [38,39]. NSEs of vaccines are not usually included in economic evaluations, but many HICs have routinely integrated vaccines’ broader epidemiological outcomes into cost-effectiveness analyses [35]. Jit and Hutubessy [37] argued that for vaccination programs funded externally, aiming to optimise global and local health benefits, the consideration of broader outcomes is justified. In LICs, where PTB burdens strain limited-resource health systems, incorporating NSE into the economic evaluation of MII may significantly impact decision-making. Given the potential underestimation of the overall benefits of MII introduction in LICs, this explorative economic evaluation aims to uncover the true value of MII, focusing on the unknown level of NSE in these settings. We incorporate a value of information (VOI) analysis to identify areas where further data could refine our understanding and decision-making regarding MII’s implementation in resource-limited settings. The study seeks to provide more economic evidence to support further clinical research into NSEs in LICs and reveal the full potential of MII.

## 2. Methods

### 2.1. Study Design

**Figure 1:**
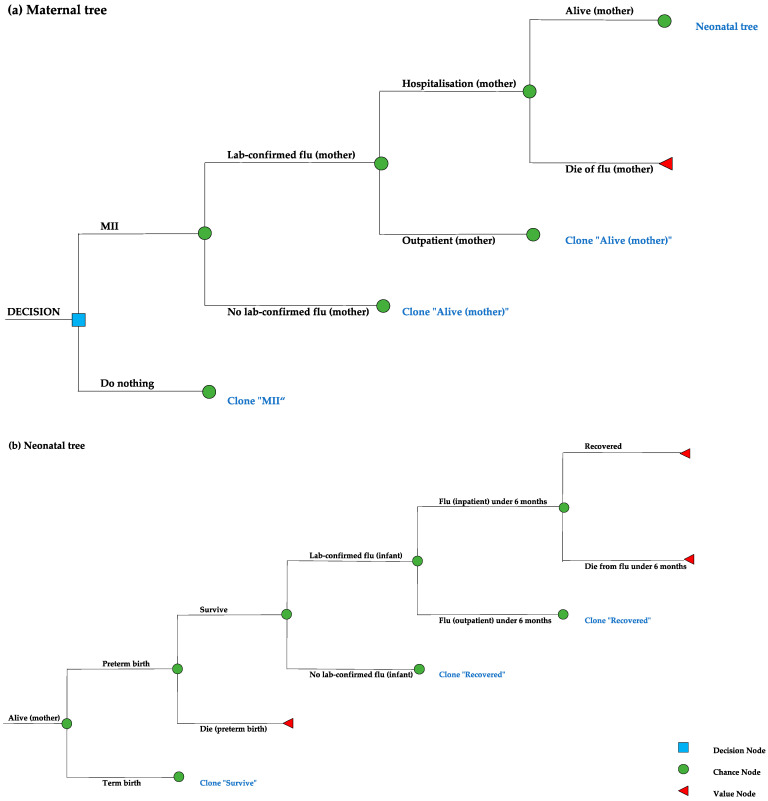
Decision tree for maternal influenza immunisation strategy (MII) and “do nothing” strategy. (**a**) Maternal tree. (**b**) Neonatal tree.

This is a modelling study conducted in a setting representative of a general low-income context, comprising all 28 countries that were categorized as ‘low-income’ by the World Bank in 2022 [40]. When data were sourced from a single study offering estimates for a general LMICs setting or a specific country, we used the study’s mean value for our base case input. When data were available from multiple individual countries, we calculated the base input by averaging these country-specific base values. Most parameters, like demographics, PTB-related outcomes and all disease management costs, were from LICs, while some parameters, such as vaccine-specific effect and influenza-related outcomes, were from LMICs. A static decision tree was built in R version 4.3.1. to estimate the cost-effectiveness of MII strategy compared to a ‘no vaccination’ strategy among pregnant women and infants under six months of age, considering both vaccine-specific effect and NSEs. As shown in Figure 1, the model captured two pregnancy outcomes, term birth and PTB, with PTB being defined as a live birth occurring before 37 weeks of gestation [17,41]. In line with WHO guidelines, we assumed that pregnant women would receive a single dose of trivalent inactivated influenza vaccine in any trimester before having a pregnancy outcome [3,10]. According to the World Bank’s population size and crude birth rates for LICs, it was estimated that approximately 25 million births occurred in LICs in 2021 [40]. To facilitate interpretation, we assumed that 100,000 pregnant women entered each strategy of our decision model. All model inputs are in Table 1.

### 2.2. Vaccine Efficacy

The model considers two types of vaccine efficacy: vaccine efficacy against influenza-related outcomes (vaccine-specific effect), and vaccine efficacy against PTB outcomes (NSE).

The vaccine-specific effect was derived from a pooled estimate from a study of three RCTs in Mali, Nepal and South Africa [24]. The study vaccine in all three countries was TIV and the types of influenza strains included H1N1 and H3N2 influenza A and influenza B. The point-estimate pooled vaccine efficacy against all influenza strains, including matched and non-matched strains, was reported as 39% (95% CI: 8–60%) in women vaccinated at any time during pregnancy and 34% (95% CI: 19–46%) in infants ≤ 6 months of age.

There has been limited clinical research investigating the NSEs of MII against PTB in LICs. Given that nearly all articles reporting positive NSEs were retrospective cohort studies, we selected model inputs based on the following selection criteria: sample size, whether immortal time bias was addressed, whether NSE was observed both within and outside the influenza season, whether the target vaccine was TIV, clarity of outcome definitions, and the methods of outcome measurement (See Appendix A Table A1). In the base case, we ran the model assuming no NSEs, reflecting the conflicting evidence around the presence of NSEs. The high value input of NSE was set at 25% based on a Canadian population cohort study with a sample size of 11,293 [17]. The study observed a protective effect of MII on adverse pregnancy outcomes inside and outside influenza seasons after addressing immortal time bias. The low value input of NSE was equivalent to the base case value of 0%.

### 2.3. Disease Burden of Influenza

We included the burden of medically attended influenza. All lab-confirmed influenza cases were assumed to be medically attended. The incidence levels of lab-confirmed influenza for pregnant women and infants under six months of age were taken from three RCTs in LMICs, with base case inputs being the pooled results [24]. Medically attended cases were divided into two main categories: outpatient and inpatient. Influenza-attributable hospitalisation rates for pregnant women were estimated based on lab-confirmed associated influenza hospitalisation incidence from a multisite cohort study in three LMICs [42]. Influenza-attributed hospitalisation rates for infants were taken from a global modelling study [5]. In medically attended influenza cases, we only included in-hospital case fatality rates due to data limitations, with inputs from a Kenya health surveillance system for pregnant women [43] and from modelling results of LMICs for infants [5]. We considered the reduced risk of infants being hospitalised for influenza given MII [44]. Of note, we were unable to capture any influenza-attributable but non-medically attended cases and death in the community due to the lack of data.

### 2.4. Disease Burden of PTB

A recent global systematic review estimated the PTB incidence for eight LICs [39]. The average PTB rate across these countries was 10%. The mortality for PTB was sourced from another global study, which provided estimates on age-standardized mortality rates for neonatal PTB (base: 13%) [45]. These data were available for 27 LICs, except for the Democratic People’s Republic of Korea. We assumed that PTB infants would not be at risk of influenza infections during their stay in the neonatal care units, and we considered the increased risk of influenza-attributed hospitalisation after discharge from neonatal care units for PTB infants [46].

### 2.5. Vaccination Coverage

Given the many barriers to the roll-out of MII and the relatively low uptake of MII in pregnant women in pilot studies in LICs [47,48], we chose a conservative vaccination coverage rate of 25% as our base case input and a range of 10–50% in sensitivity analysis. We assumed all pregnant women in the cohort would not be covered by other influenza vaccine programs. 

### 2.6. Cost and Health Resources Use

We captured vaccination program costs (i.e., vaccine dose price and vaccine delivery cost) and disease management costs (i.e., costs of outpatient visits and inpatient days). All costs were adjusted to 2021 United States dollars (USD) following the approach recommended in Turner et al. [49]. As there were no procurement records of TIV (adults) for LICs in the WHO MI4A 2022 Public Database, the average TIV dose price (USD 3.7) in 2021 for lower-MICs was used, regardless of sponsorship and procurement method [28]. Vaccine delivery costs were economic costs from a Malawian study, which reflected opportunity costs [47]. The base delivery cost was set at USD 7.3, corresponding to a coverage level of 25%. For the sensitivity analysis, the low value was USD 5.8 reflecting 50% coverage, and we assumed a high value of 1.2 times the base case value for 10% coverage. We include a 10% vaccine wastage rate in the base case based on studies from Gambia and WHO (varied from 0 to 20% in sensitivity analyses) [50,51]. 

All outpatient visits and inpatient bed day unit costs were from WHO-CHOICE [52]. For influenza outcomes, we considered both outpatient visit and inpatient day costs. We assumed one outpatient visit per laboratory-confirmed influenza case. The cost for hospitalised influenza per case was calculated as the daily inpatient bed day unit cost multiplied by length of stay, which we estimated to average 4 days, with a range of 1–7 days [42]. 

For PTB, we considered the cost of inpatient neonatal intensive care for PTB. Typically, the cost of neonatal intensive care is substantially higher than general ward costs, often by several folds [53]. However, this cost differential may be reduced due to the less intensive nature of services, a consequence of limited resources in LMICs [53]. Therefore, we conservatively assumed the cost of neonatal care per day to be twice the WHO-CHOICE inpatient unit cost. Drawing from a clinical study in Gambia [54], we estimated that the average length of stay for preterm survivors was 16 days, ranging from 6 to 26 days. For those who died despite receiving care, the average stay was 4 days, within a range of 1–6 days. Given the resource constraints in low-income countries, we considered that not all neonates would have access to healthcare services. Therefore, we estimated the proportion of preterm infants accessing neonatal care based on non-home-birth delivery rates in LICs (proportion of access to healthcare services = 1 − home-birth delivery rates in LICs) [55]. In the base case, the proportion was 63%.

We excluded the costs of medications and examinations due to the challenges in finding uniform costing sources and to avoid an overestimation of costs (e.g., medication and radiology may not be needed for all influenza cases in LICs).

### 2.7. Health Outcomes

The main outcome measure was years of life lost (YLLs) averted and other outcomes included adverse case averted (i.e., influenza and PTB). The YLLs due to premature death of pregnant women from influenza was estimated based on the average age of pregnant women and the health-adjusted life expectancy of the population in the sub-Sahara region [56,57]. The YLLs for neonatal death was assumed to be the same as the health-adjusted life expectancy [56]. We did not capture years lived with a disability as the reliable prevalence and duration of influenza infection data are scarce in LICs, due to the underreporting of cases in community settings. 

### 2.8. Cost-Effectiveness Analysis

All analyses were from the perspective of healthcare systems, considering all payers. The time horizon of the decision tree was two years, which aligns with the critical period during which maternal and neonatal outcomes are observed [58]. An annual discount rate of 3% was applied to future costs and YLLs according to WHO guidance [58]. We did not include side-effects such as redness and swelling after the injection as this is unlikely to affect the cost-effectiveness [34,58]. We assumed that all pregnancies were singleton. We applied country-specific 2021 GDP per capita to estimate opportunity-cost based cost-effectiveness thresholds (CETs) for all available LICs based on a global study from Pichon-Riviere et al. [59]. The average CET across LICs was USD 188, with a range from USD 28 to USD 538. The analyses were performed according to WHO’s Guidance on the Economic Evaluation of Influenza Vaccination [58].

### 2.9. Sensitivity Analysis

Different sensitivity analyses were carried out to investigate the uncertainty of model inputs and in particular how the cost-effectiveness changed across different levels of NSE inputs. We conducted one-way sensitivity analysis (OWSA) and multi-way sensitivity analysis (MWSA), where we changed one or several model inputs in their predetermined range to examine changes in cost effectiveness. Probabilistic sensitivity analysis (PSA) was used to assess the robustness of the study results, with 10,000 iterations of Monte Carlo simulations where all model inputs were under a specific distribution (see distributions in Table 1). 

### 2.10. Value of Information Analysis

We conducted a VOI to assess the potential benefits of additional research in reducing uncertainty in decision making [60,61]. Employing the expected value of perfect information (EVPI), we explored the full potential value of eliminating all uncertainties within our model’s inputs. The expected value of partial perfect information (EVPPI) was performed to isolate the value of resolving uncertainties related to NSE on PTB. Additionally, we utilized the expected value of sample information (EVSI) to understand how the expected value of further research fluctuated with changes in sample size. Using the EVPPI, we quantified the maximum investment for resolving NSE on PTB uncertainties for a population of 100,000 in each LIC, tailoring this estimation to country-specific CETs. Since our model synthesized various sources of inputs and combined them in a complex and nonlinear way, we used Gaussian process regression (a nonlinear method) to conduct VOI [62].

**Table 1:** Model input parameter values for base case and sensitivity analysis.

Inputs	Base Value	Low Value	High Value	Distribution	Reference
*Vaccine specific efficacy (against incidence of influenza)*					
Pregnant women	39%	8%	60%	Beta	LMICs study [24]
Infants under 6 months of age	34%	19%	46%	Beta	LMICs study [24]
*Vaccine non-specific efficacy*					
Preterm birth	0%	0%	25%	Uniform (0–5% in PSA)	Base case conservative assumption based on existing conflicting evidence and high value from Canadian study [17]
*Burden of influenza illness*					
Incidence attack rate, lab-confirmed (pregnant women)	1.18%	0.82%	3.60%	Beta	LMICs study [24]
Incidence attack rate, lab-confirmed (infants)	4.36%	0.49%	5.53%	Beta	LMICs study [24]
Hospitalisation rate given influenza (pregnant women)	2.34%	1.80%	5.18%	Beta	Estimated based on LMICs study [42]
Hospitalisation rate given influenza (infants)	1.30%	0.18%	3.10%	Beta	LMICs in Global study [5]
In-hospital case fatality rate of influenza (pregnant women)	6.80%	1.10%	12.50%	Beta	Kenyan study [43]
In-hospital case fatality rate of influenza (infants)	3.20%	0.60%	15.40%	Beta	LMICs in Global study [5]
Reduced risk of influenza-attributed hospitalisation for infants given maternal immunisation	0.61	0.45	0.84	Log normal	US study [44]
Increased risk of influenza-attributed hospitalisation for preterm birth infants	2.24	1.44	3.5	Log normal	Norwegian study [46]
*Burden of preterm birth*					
Preterm birth incidence	10%	3%	22%	Beta	LICs in global study [39]
Preterm birth mortality	13%	3%	34%	Beta	LICs in global study [45]
Proportion of preterm infants receiving neonatal care	62%	20%	94%	Beta	Estimated based on LMIC study [55]
*Vaccine coverage and wastage*					
Vaccine coverage	25%	10%	50%	Beta	Malawian study [47]
Vaccine wastage	10%	0%	20%	Beta	Estimated based on WHO and Gambian study [50,51]
*Costs of vaccination program*					
Trivalent influenza vaccine dose price	USD 3.7	USD 3.3	USD 4.4	Beta	WHO database [28]
Vaccine delivery cost	USD 7.3	USD 5.8	USD 8.7	Beta	Estimated based on Malawian study [47]
*Cost of disease management*					
Outpatient visits for influenza per case	USD 5.5	USD 0.4	USD 19.2	Gamma	Estimated based on WHO-CHOICE for all available LICs
Hospitalisation for influenza per case	USD 79.8	USD 1.7	USD 478.4	Gamma	Estimated based on WHO-CHOICE for all available LICs
Neonatal care for preterm infants (survived) per case	USD 638.4	USD 19.8	USD 3553.7	Gamma	Estimated based on WHO-CHOICE for all available LICs
Neonatal care for preterm infants (died) per case	USD 159.6	USD 3.3	USD 820.1	Gamma	Estimated based on WHO-CHOICE for all available LICs
*Utilities*					
Years of life lost, maternal death	28.7	25.7	30.7	Log normal	Sub-Saharan region in global study and UN report [56,57]
Years of life lost, neonatal death	57.4	54.8	59.8	Log normal	Sub-Saharan region in global study [56]

LICs = low-income countries; LMICs = low- and middle-income countries; US = United States; USD = United States dollars; UN = United Nations. Note: in cases where data were available from multiple countries, we calculated our model’s base input by averaging the base or mean estimates from each of these countries. For the high/low inputs, we selected the single highest/lowest value among all countries, rather than averaging the high and low estimates across countries.

## 3. Results

### 3.1. Base Case Results

Table 2 and Table 3 present the 3% discounted costs and health benefits of introducing MII to 100,000 pregnant women in LICs over a two-year time horizon. Across different NSE levels, neonates’ YLLs averted consistently accounted for over 90% of the total YLLs averted. Regardless of NSE levels, MII introduction would incur an additional vaccine-related cost of USD 284,098. At the base NSE level (0%), MII incurred additional costs amounting to USD 272,607 with relatively small health benefits of 84 YLLs. This resulted in an ICER of USD 3262, which is unlikely to be cost-effective in LICs in the absence of NSE. At increasing NSE levels of 5%, 10%, and 25%, the number of PTBs and therefore neonatal YLLs averted increased. When considering a small NSE level (5%), MII introduction may be cost-effective, averting 3600 YLLs with an ICER of USD 28, well below the base CET of USD 188. At higher NSE levels of 10% to 25%, MII introduction dominates the ‘do nothing’ strategy as it prevents more adverse pregnancy outcomes associated with costly health services. 

### 3.2. Sensitivity Analyses

#### 3.2.1. One-Way Sensitivity Analysis

**Figure 2:**
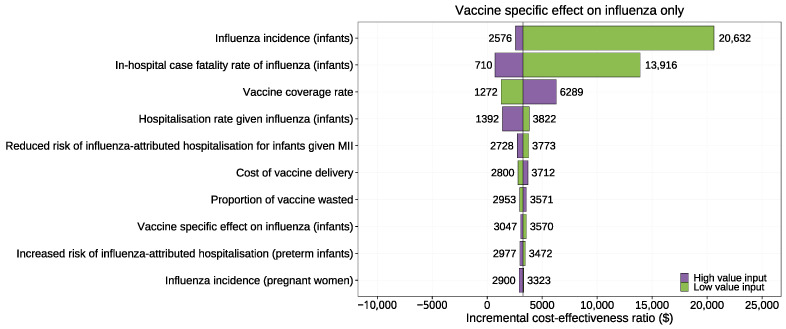
Tornado plot for one-way sensitivity analysis considering vaccine-specific effect on influenza only.

The OWSA was applied for all model inputs, yet herein we report on the top ten most sensitive inputs. Our OWSA incorporated scenarios both with and without the consideration of NSEs on PTB. Figure 2 shows the base case scenario where only vaccine-specific effects on influenza outcomes were considered. The most influential inputs were neonatal influenza outcomes, such as influenza incidence and in-hospital case fatality rates. In the absence of any NSEs, the variability of any single input was insufficient to establish the cost-effectiveness of the MII strategy in any LIC, suggesting that when considering only vaccine-specific effect, the MII strategy was unlikely to be cost-effective in LICs. 

In addition to examining vaccine-specific effect, we also explored scenarios considering both vaccine-specific effects and non-specific effects (NSEs), exploring values for NSEs of 5% and 10% (range: 0–25%). The results suggested that cost-effectiveness was most influenced by PTB-related outcomes including NSEs on PTB, cost of neonatal care for PTB survivors, PTB mortality rate and proportion of PTBs receiving neonatal care. Apart from the value of NSE itself, variations in any other single input consistently resulted in MII strategy being either dominating or cost-effective in LICs (where NSEs were held at 5% or higher).

#### 3.2.2. Multi-Way Sensitivity Analysis

**Figure 3:**
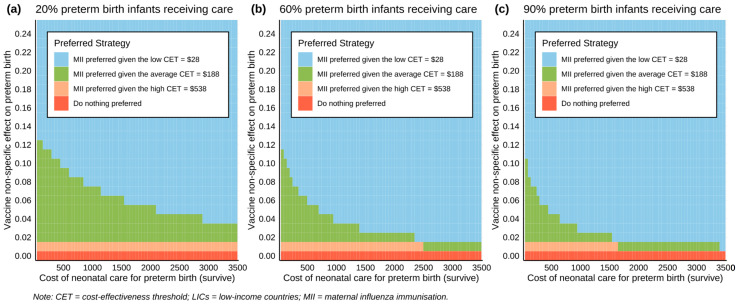
Multi-way sensitivity analysis plots: (**a**–**c**) show combined effects of non-specific effect input and cost of preterm birth care input under low/medium/high proportions of preterm infants’ access to care.

Within the MWSA, the combined effect of simultaneous variations in the three highly sensitive and most uncertain inputs (NSEs on PTB, cost of neonatal care for PTB survivors and proportion of PTBs receiving neonatal care) was explored. Figure 3 revealed that MII strategy was preferred in most scenarios when multiple inputs of high uncertainty were varied concurrently. The ‘do nothing’ strategy was only favoured when NSEs on PTB were at the minimum or absent. It is worth noting that even a small NSE on PTB (about 5%) could make MII a cost-effective strategy at average CET in LICs, regardless of the variation in cost of neonatal care for PTB. Moreover, as the proportion of PTBs receiving care increased, MII strategy would be increasingly favourable at lower CETs.

#### 3.2.3. Probabilistic Sensitivity Analysis

**Figure 4:**
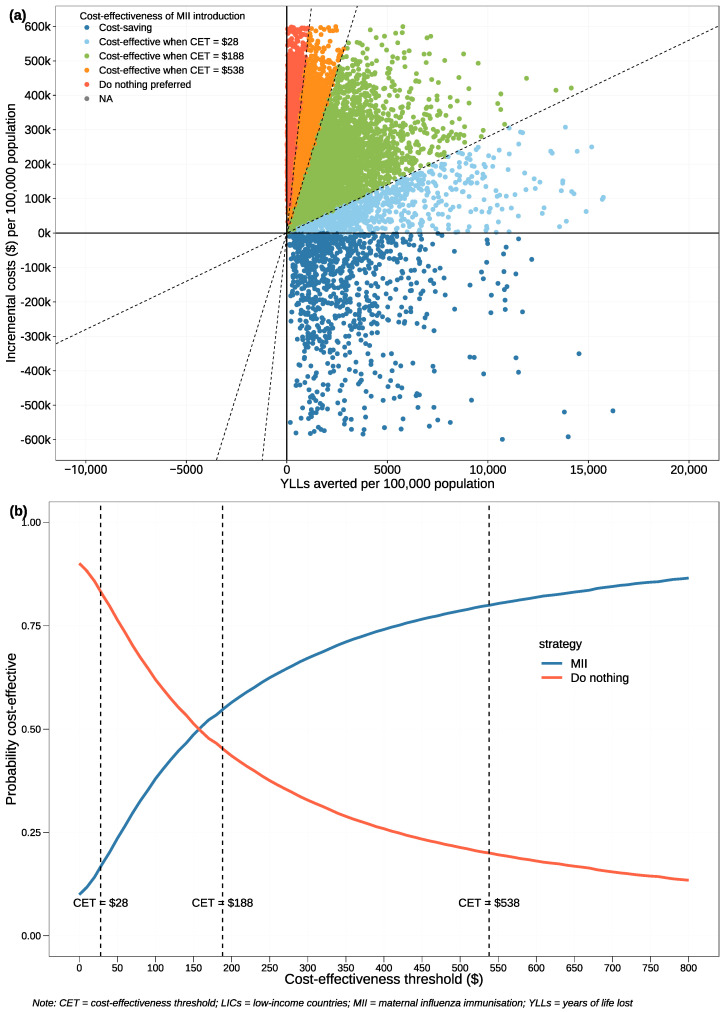
Probabilistic sensitivity analysis plots assuming 0–5% non-specific effects on preterm birth with uniform distribution. (**a**) Cost-effectiveness plane for 10,000 iterations. (**b**) Cost-effectiveness acceptability curve across different cost-effectiveness thresholds.

For the PSA, we limited the NSEs on PTB to a range of 0–5%, following a uniform distribution. This small range was selected based on prior results, suggesting that a broader range would further skew results in favour of MII strategy. A uniform distribution was employed for this input to accommodate the possibility of a zero effect, which is a realistic consideration and cannot be represented with a beta distribution. The cost-effectiveness plane illustrates that most iterations fell within the CETs applicable to LICs (Figure 4a). The CEAC depicted in Figure 4b shows that MII had a probability of approximately 55% of being cost-effective under the base CET of USD 188. At the low CET of USD 28, the probability that MII was cost-effective diminished, reflecting greater uncertainty in its economic justification without significant NSEs. When the CET was raised to USD 538, the probability that MII was the preferred strategy approached 80%.

### 3.3. Value of Information Analysis

**Figure 5:**
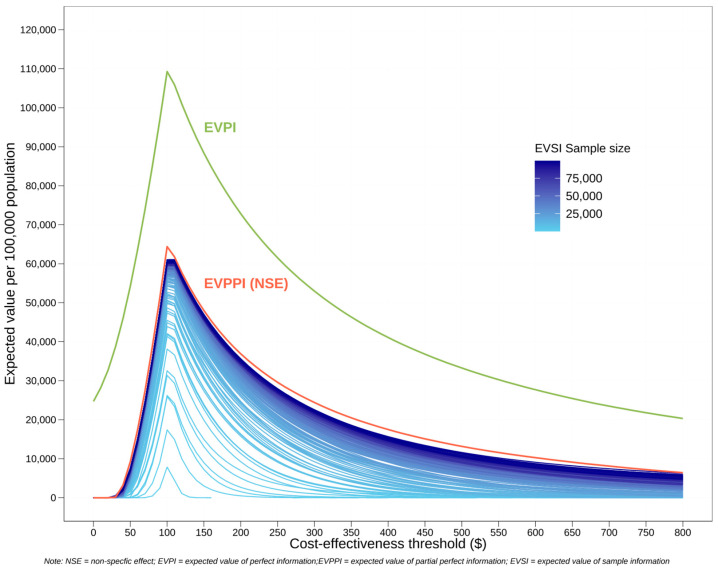
Value of information analysis plot. The red curve is the expected value of partial perfect information when conditioning on “non-specific effect on preterm birth” (0–5% with uniform distribution). The green curve is the expected value of perfect information for eliminating uncertainties of all model inputs. Each blue curve corresponds to a sample size. The sample size ranges from 100 to 100,000. The larger the sample size, the darker the colour of the curve.

As shown in Figure 5, both EVPI and EVPPI (condition on NSEs on PTB) peaked at CETs of around USD 100–125. This peak reflected substantial decision uncertainty in cost-effectiveness at below-average CET levels, suggesting that additional information on all model inputs or on NSEs only could be highly valuable in these instances. Based on the PSA, the current decision at CETs around USD 100 favoured the ‘do nothing’ strategy. However, the acquisition of additional information had the potential to shift this decision. It also showed that the EVPI consistently exceeded the EVPPI, which was expected since the uncertainties associated with all model inputs combined would be naturally greater than that for NSEs on PTB alone. Notably, the value of EVPPI was quite significant, approximating half the value of EVPI, underscoring that the uncertainty regarding NSEs on PTB alone could be a major contributor to the overall uncertainty in the model.

Given our intention to conduct further research on NSEs, we estimated the EVSI of a series of different sample sizes for this study. The EVSI approached the EVPPI at larger sample sizes, indicating that the value of information gained from larger studies may approach the theoretical maximum value of eliminating the uncertainty of NSE on PTB. However, the closeness of EVSI to EVPPI also suggested that beyond a certain sample size, the additional value of information began to plateau. The incremental gain from increasing the sample size further became marginal, implying that while large-scale studies can significantly reduce uncertainty, they may not always be the most cost-effective use of resources.

**Figure 6:**
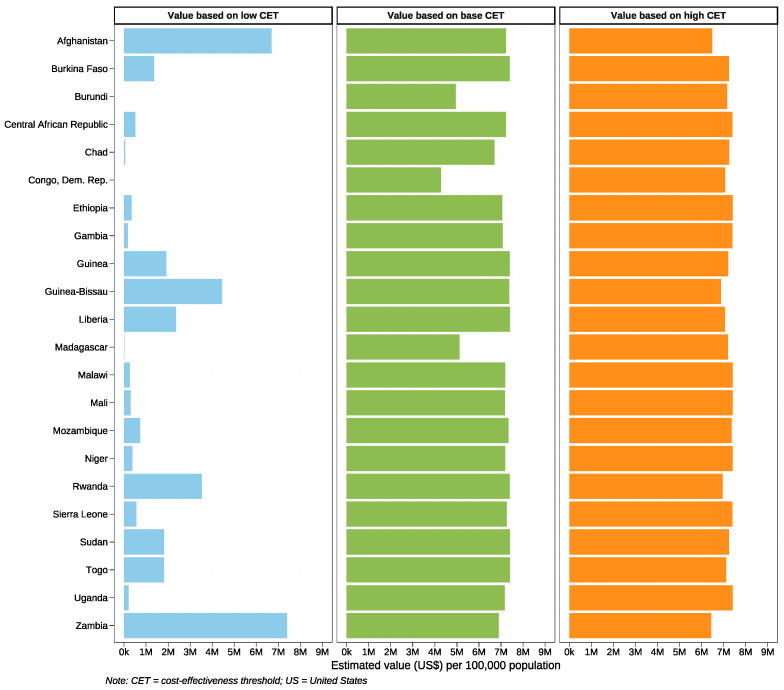
Estimated population value (USD) of further research on maternal influenza immunisation non-specific effects on preterm birth in 22 low-income countries based on country-specific base/low/high cost-effectiveness thresholds. Assume the research period is one-year. The countries where the cost-effectiveness threshold value is unavailable have been removed from this plot.

We then estimated the value of conducting further research on NSEs based on CETs for individual LICs. This was estimated by multiplying the population EVPPI with country-specific CETs. Certain countries were not included in the evaluation due to the unavailability of reported CETs. The estimated value of additional research on NSEs regarding PTB for a population of 100,000 was depicted in Figure 6, reflecting the value for a single year. This population can be adjusted proportionally based on different sample sizes, and for multi-year studies, the values would be discounted to the present value, using an appropriate discount rate to reflect the time value of money.

Taking the example of Mali, where the estimated value based on the base CET was USD 7 million per 100,000 population annually, let us consider the scenario for conducting a cohort study. If we aim to collect NSE evidence with a study sample size of 10,000, and the research spans 3 years with an annual discounting rate of 3%, the total discounted value for the study would be calculated as follows:

(1) Total Costs=∑n=137,000,000×(10,000/100,000) 1+0.03^n = USD 1,980,028

Therefore, the estimated research value for conducting such a study in Mali with a sample size of 10,000, over a period of 3 years with a 3% annual discount rate, is approximately USD 1.98 million. If the budget for this research does not exceed the estimated values depicted, then the pursuit of further research may be considered cost-effective, as the value of the additional information gained would justify the investment.

## 4. Discussion

Our model results suggest that MII introduction may be cost-effective or cost-saving in LICs when the level of NSE on PTB is at least 5%. NSE is the main driver of cost-effectiveness and thus understanding the magnitude of these effects, and whether they are likely to exist in LIC settings, is important. The PSA further revealed the possibility that even a small NSE on PTB could tip the balance in favour of MII being cost-effective in LICs. The protective effect observed in NSE studies, which were included in our literature review, primarily ranged between 30% and 40%. This could explain why the MII strategy was estimated to be dominating when the NSE level was 10–25% in the base case analysis, as the magnitude of NSEs significantly exceeded a “small” effect. The VOI analysis conducted as part of our study provided insights into the potential benefits of further research on the NSEs of MII in LICs. The VOI analysis demonstrated that additional research to elucidate the magnitude of NSEs could greatly influence the cost-effectiveness evaluation of the MII strategy.

Our study’s methodology exhibits several strengths that enhance its relevance and applicability to low-income contexts. A key aspect of our approach was the comprehensive use of data from all available LICs to represent the general situation in these regions. This approach ensures that the findings are broadly applicable and not skewed by data from a select few countries. Our strategic use of estimates from global studies, LMICs studies and the WHO database ensures consistent and representative methodologies across all LICs. We acknowledge the significance of opportunity costs in decision-making within LICs. Accordingly, our analysis used vaccine delivery costs and CETs that account for opportunity costs. This ensures a more realistic assessment of economic implications, aligning closely with the resource-constrained realities of these settings.

Our research stands out as one of the few studies examining the cost-effectiveness of MII in LICs. A key distinction from the only prior LIC-focused study is our consideration of NSEs. Of note, when only considering vaccine-specific effects, our findings were consistent with the previously published study that found that MII was not cost-effective in LICs. However, our estimated ICER exceeded USD 3000, substantially higher than previous findings (base ICER = USD 847) [33]. This discrepancy was likely due to our use of more conservative pooled estimates for vaccine efficacy, approximately half of those used in the prior study, and our vaccine delivery costs, which were sevenfold higher after accounting for opportunity costs. Therefore, our analysis presents a more conservative estimate, even when restricted to consider only vaccine-specific effects.

Our study had several limitations. Most critically, the presence and level of NSEs on PTB remains uncertain. While there are some data from LICs indicating an absence of beneficial NSEs on PTB by MII, it is important to recognise that these studies were not designed to evaluate PTB outcomes, but were set up with influenza-related outcomes as their primary outcomes of interest. The only specifically NSE-focused MII studies available were from observational studies from HICs, which may not reflect the observed protection in an LIC setting [13,14]. This also highlights the need for more evidence on NSEs of MII in LICs. Secondly, our VOI analysis, while assessing EVPPI for country-specific CETs, did not delve into a comprehensive country-specific analysis using all model inputs from a certain country. This limitation was due to the unavailability of complete input data for each LIC. For future clinical research on NSEs targeted at a specific country, a re-assessment using country-specific data would ensure a more accurate evaluation of the potential benefits of additional research in that specific context. Thirdly, the model could potentially undervalue the overall benefits of introducing MII, as it may not fully capture the economic and disease burden, leading to a conservative estimate of its benefits. We also did not account for years lived with a disability, thereby neglecting the potential disability-adjusted life years lost from influenza and PTB. The disease burden of seasonal influenza was expected to be substantial in LICs due to weaker health systems, lower vaccination coverage, and high population density [63,64]. Nonetheless, it is still difficult to estimate the actual burden of seasonal influenza, as fewer data were documented in LICs [63]. Additionally, we may underestimate the economic burden of diseases. We did not capture any cost of productivity loss due to seasonal influenza, which may be expected to be high in LICs [65]. PTBs can incur greater costs from a societal perspective due to their indirect and intangible costs to the community [66]. However, we were unable to obtain those cost data due to limited evidence. The static nature of the model may also be a limitation. Although the WHO’s guidance suggests that a static model is adequate in most cases of influenza vaccination economic evaluations, it was unable to capture dynamic changes, like yearly variations in circulating influenza, viral versus vaccine strain matching and seasonality.

Given the high burden of PTB in LICs, this study emphasizes the need for future clinical research to include NSEs of MII in these settings. If NSEs are confirmed then MII may be cost effective and lead to renewed decision making regarding MII introduction.

## 5. Conclusions

Incorporating the possibility of NSEs on PTB into our analysis suggests that MII introduction in LICs could be cost-effective or cost-saving. Notably, even a small NSE (as low as 5%) could significantly influence decision-making in this context. Existing evidence of a protective effect of maternal influenza vaccination against PTB remains inconclusive, so further empirical research would be of value to comprehensively understand the potential presence of NSEs of MII to guide public health policy in low-income settings.

## Figures and Tables

**Table 2:** Estimated maternal and neonatal health benefits of MII per 100,000 population in LICs across different NSE levels on PTB.

Health Outcome	NSE Level	Do Nothing	MII	Averted
Total life years lost	0%	70,492	70,408	84
	5%	-	66,892	3600
	10%	-	67,186	3306
	25%	-	52,824	17,668
Neonate life years lost	0%	70,441	70,363	79
	5%	-	66,846	3595
	10%	-	67,186	3256
	25%	-	52,778	17,663
Maternal life years lost *	0–25%	51	46	5
Influenza cases, mothers *	0–25%	1180	1065	115
Influenza cases, infants	0%	4303	2840	1463
	5%	-	2842	1461
	10%	-	2844	1459
	25%	-	2850	1454
Preterm birth cases	0%	10,000	10,000	0
	5%	-	9500	500
	10%	-	9000	1000
	25%	-	7500	2500
Preterm birth-attributable death	0%	1300	1300	0
	5%	-	1235	65
	10%	-	1170	130
	25%	-	975	325

LICs = low-income countries; MII = maternal influenza immunisation; NSE = non-specific effect; PTB = preterm birth; “-” indicates that the results of “do nothing” are the same as the 0% non-specific effect level. * Values are constant across all non-specific effect levels.

**Table 3:** Estimated costs and cost-effectiveness ratios of MII per 100,000 population in LICs.

	NSE Level	Do Nothing	MII	Incremental
Total costs	0%	3,455,853	3,728,460	272,607
	5%	-	3,557,395	101,542
	10%	-	3,386,329	−69,524
	25%	-	2,832,403	−623,450
Vaccine-related costs *	0–25%	0	284,098	284,098
Influenza-related costs	0%	34,447	22,949	−11,498
	5%	-	22,955	−11,493
	10%	-	22,960	−11,487
	25%	-	22,977	−11,470
Preterm birth care costs	0%	3,421,406	3,421,412	6
	5%	-	3,250,342	−171,064
	10%	-	3,079,270	−342,136
	25%	-	2,566,059	−855,347
ICERs (Total life years lost)	0%	Reference	3262	
	5%	Reference	28	
	10%	Reference	Dominating	
	25%	Reference	Dominating	

ICERs = incremental cost-effective ratios; LICs = low-income countries; MII = maternal influenza immunisation; NSE = non-specific effect; “-” indicates that the results of “do nothing” are the same as the 0% non-specific effect level. * Values are constant across all non-specific effect levels.

## Data Availability

The data supporting the findings of this study are available within the article/Appendix. Further inquiries can be directed to the corresponding author.

## Appendix A

**Table A1:** Maternal influenza immunisation positive non-specific effect studies.

Country	Year	Sample Size	Study Type	Result	Outcome	Things to Consider	Reference
Canada	2012	55,570	Retrospective Cohort Study	aRR [95% CI]: 0.73 [0.58, 0.91]	Preterm birth (<37 weeks)	H1N1; lack of time-varying exposure analysis	[22]
Australia	2021	269,493	Retrospective Cohort Study	aRR [95% CI]: 0.69 [0.66, 0.72]	Preterm birth (<37 weeks)	Not TIV	[15]
Canada	2014	11,293	Retrospective Cohort Study	aRR [95% CI]: 0.75 [0.60, 0.94]	Preterm birth (<37 weeks)	TIV; time-varying analysis; sub-group analysis	[17]
South Africa	2021	4084	Retrospective Cohort Study	aOR [95% CI]: 0.61 [0.44, 0.85] (primigravidae)	Preterm birth (<37 weeks)	Lack of time-varying exposure analysis	[16]
Lao	2016	5103	Retrospective Cohort Study	RR [95% CI]: 0.56 [0.45, 0.70]	Preterm birth (<37 weeks)	The sample size outside flu season may be too small to detect differences in PTB rates	[19]
Nicaragua	2017	3258	Retrospective Cohort Study	aOR [95% CI]: 0.66 [0.45, 0.96] (vaccinated in third trimester)	Preterm birth (<37 weeks)	Potential selection bias and recall bias	[20]
USA	2012	10,225	Retrospective Cohort Study	5% vs. 6% in vaccinated and unvaccinated group	Preterm birth (<37 weeks)	Lack of time-varying exposure analysis	[21]
Multiple	2019	40 studies	Systematic Review	aOR [95% CI]: 0.87 [0.78, 0.96]	Preterm birth (<37 weeks)	Not TIV	[15]

aRR = Adjusted risk ratio; aOR = adjusted odds ratio; CI = confidence interval; QIV = quadrivalent inactivated influenza vaccines; TIV = trivalent influenza vaccines.

**Table A2:** Country-specific cost-effectiveness thresholds (2021 USD).

Country	2021 GDP per Capita ^1^	Base CET ^2^	Low CET ^2^	High CET ^2^
Afghanistan	517	336	103	527
Burkina Faso	918	211	64	331
Burundi	237	92	28	144
Central African Republic	512	153	51	246
Chad	696	104	35	160
Democratic People’s Republic of Korea	NA	NA	NA	NA
Democratic Republic of the Congo	584	88	29	134
Eritrea	643	NA	NA	NA
Ethiopia	944	132	47	217
Gambia	836	134	42	209
Guinea *	1174	211	70	341
Guinea-Bissau	813	276	89	431
Liberia	673	249	74	384
Madagascar	515	93	31	149
Malawi	643	148	45	231
Mali	918	147	46	229
Mozambique	500	180	55	280
Niger	595	149	48	232
Rwanda	834	258	83	409
Sierra Leone	516	160	52	253
Somalia	446	NA	NA	NA
South Sudan	1120	NA	NA	NA
Sudan	764	214	69	329
Syrian Arab Republic	1266	NA	NA	NA
Togo	992	238	69	367
Uganda	858	146	43	232
Yemen	691	NA	NA	NA
Zambia *	1121	426	213	538

CET = Cost-effectiveness threshold; NA = not available; ^1^ 2021 GDP per capita data from the World Bank; ^2^ CETs were based on Pichon-Riviere et al. [59], adjusted by 2021 GDP per capita; * Guinea and Zambia were not classified as “low-income” in 2023 based on the World Bank, but we included them in our study as they were low-income countries during our study period.

**Table A3:** Preterm birth incidence rate in low-income countries (%).

Country	Base	Low	High
Democratic Republic of the Congo	12.4	7.8	19.5
Ethiopia	12.9	7.9	20.7
Malawi	14.5	9.5	21.6
Mali	6.1	3.1	11.5
Mozambique	7.0	4.4	10.9
Rwanda	9.3	3.7	21.2
Uganda	10.0	6.1	16.0
Zambia	7.7	4.5	12.5

Source: the estimates of 2020 preterm rate from Ohuma et al. [39].

**Table A4:** Preterm birth neonates’ mortality in low-income countries (%).

Country	Base	Low	High
Afghanistan	12.0	6.3	19.6
Burkina Faso	13.7	9.1	19.5
Burundi	10.1	6.7	14.3
Central African Republic	23.5	16.0	32.9
Chad	16.3	10.1	22.9
Democratic People’s Republic of Korea	NA	NA	NA
Democratic Republic of the Congo	12.3	8.0	17.3
Eritrea	8.2	4.8	12.9
Ethiopia	9.2	6.5	12.4
Gambia	10.2	5.7	16.8
Guinea	15.9	10.7	21.9
Guinea-Bissau	18.6	13.0	25.1
Liberia	12.1	7.8	17.9
Madagascar	12.5	8.4	17.6
Malawi	9.9	6.2	14.3
Mali	23.1	15.8	32.0
Mozambique	11.6	7.7	16.3
Niger	14.2	10.2	18.9
Rwanda	8.6	5.6	12.0
Sierra Leone	17.5	12.1	24.5
Somalia	11.9	7.5	18.1
South Sudan	14.6	9.6	20.4
Sudan	25.2	18.4	34.0
Syrian Arab Republic	4.0	2.6	5.7
Togo	13.7	9.7	18.0
Uganda	5.2	3.5	7.5
Yemen	22.9	14.8	32.7
Zambia	7.5	4.6	11.2

NA = Not available. Source: the estimates of age-standardized mortality rates of neonatal preterm birth in 2019 from Cao et al. [45].

**Table A5:** Cost per outpatient visit in low-income countries (2021 USD).

Country	Base ^1^	Low ^2^	High ^3^
Afghanistan	NA	NA	NA
Burkina Faso	13.0	9.9	14.5
Burundi	0.6	0.4	0.6
Central African Republic	15.3	11.6	17.0
Chad	17.2	13.1	19.2
Democratic People’s Republic of Korea	NA	NA	NA
Democratic Republic of the Congo	NA	NA	NA
Eritrea	NA	NA	NA
Ethiopia	1.2	0.9	1.4
Gambia	1.7	1.3	1.8
Guinea	1.8	1.4	2.0
Guinea-Bissau	12.8	9.7	14.2
Liberia	NA	NA	NA
Madagascar	0.8	0.6	0.9
Malawi	1.0	0.8	1.1
Mali	12.1	9.2	13.5
Mozambique	1.1	0.8	1.2
Niger	7.6	5.7	8.4
Rwanda	1.2	0.9	1.3
Sierra Leone	1.1	0.8	1.2
Somalia	NA	NA	NA
South Sudan	NA	NA	NA
Sudan	2.2	1.6	2.4
Syrian Arab Republic	NA	NA	NA
Togo	10.7	8.1	11.9
Uganda	1.4	1.1	1.5
Yemen	NA	NA	NA
Zambia	1.2	0.9	1.3

NA = Not available. This can be due to the data not being included in the WHO-CHOICE or the local inflation index being unavailable. Source: WHO-CHOICE. All costs are inflated from the 2010 local currency unit to 2021 based on the local consumer price index in the International Monetary Fund, and then converted from local currency to U.S. dollars based on the exchange rate. ^1^ Base value is the average cost across all primary health facilities in a country. ^2^ Low value is the lowest cost among all primary health facilities in a country. ^3^ High value is the highest cost among all primary health facilities in a country.

**Table A6:** Cost per inpatient day in low-income countries (2021 USD).

Country	Base ^1^	Low ^2^	High ^3^
Afghanistan	NA	NA	NA
Burkina Faso	43.9	45.8	59.2
Burundi	1.5	1.5	2.0
Central African Republic	44.3	46.1	59.7
Chad	60.5	63.0	81.5
Democratic People’s Republic of Korea	NA	NA	NA
Democratic Republic of the Congo	NA	NA	NA
Eritrea	NA	NA	NA
Ethiopia	3.8	4.0	5.1
Gambia	6.4	6.7	8.7
Guinea	5.6	5.8	7.5
Guinea-Bissau	41.9	43.7	56.5
Liberia	NA	NA	NA
Madagascar	2.4	2.5	3.2
Malawi	2.9	3.1	4.0
Mali	40.4	42.1	54.5
Mozambique	3.2	3.3	4.3
Niger	20.3	21.1	27.3
Rwanda	3.9	4.0	5.2
Sierra Leone	3.5	3.7	4.8
Somalia	NA	NA	NA
South Sudan	NA	NA	NA
Sudan	9.1	9.5	12.3
Syrian Arab Republic	NA	NA	NA
Togo	32.8	34.2	44.2
Uganda	4.7	4.9	6.3
Yemen	NA	NA	NA
Zambia	4.3	4.5	5.8

NA = Not available. This can be due to the data not being included in the WHO-CHOICE or the local inflation index being unavailable. Source: WHO-CHOICE. All costs are inflated from the 2010 local currency unit to 2021 based on the local consumer price index in the International Monetary Fund, and then converted from local currency to U.S. dollars based on the exchange rate. ^1^ Base value is the average cost across all hospital levels in a country. ^2^ Low value is the lowest cost among all hospital levels in a country. ^3^ High value is the highest cost among all hospital levels in a country.

**Table A7:** Proportion of preterm infants accessing neonatal care.

Country	Base	Low	High
Afghanistan	49%	45%	53%
Burkina Faso	67%	64%	70%
Burundi	88%	87%	89%
Central African Republic	NA	NA	NA
Chad	23%	20%	25%
Democratic People’s Republic of Korea	NA	NA	NA
Democratic Republic of the Congo	81%	78%	84%
Eritrea	NA	NA	NA
Ethiopia	27%	24%	31%
Gambia	63%	59%	68%
Guinea	53%	49%	56%
Guinea-Bissau	NA	NA	NA
Liberia	56%	53%	60%
Madagascar	36%	33%	39%
Malawi	93%	92%	94%
Mali	67%	63%	71%
Mozambique	57%	53%	60%
Niger	30%	28%	33%
Rwanda	92%	91%	93%
Sierra Leone	84%	81%	86%
Somalia	NA	NA	NA
South Sudan	NA	NA	NA
Sudan	NA	NA	NA
Syrian Arab Republic	NA	NA	NA
Togo	73%	70%	77%
Uganda	75%	73%	77%
Yemen	31%	29%	33%
Zambia	85%	83%	87%

NA = Not available. Source: we estimated the proportions based on the home birth rates in LMICs from Hernández-Vásquez et al. [55]. The proportion of accessing neonatal care is 1—home birth rate.
